# IL‐17 and IL‐23 inhibitors dose spacing in adult psoriatic patients: a real‐world pilot study

**DOI:** 10.1111/ddg.15686

**Published:** 2025-04-26

**Authors:** Luca Mastorino, Paolo Dapavo, Michela Ortoncelli, Eleonora Bongiovanni, Pietro Quaglino, Simone Ribero

**Affiliations:** ^1^ Dermatologic Clinic Department of Clinical Medicine University of Turin Turin Italy

**Keywords:** Psoriasis, IL‐23 inhibitors, IL‐17 inhibitors, dose spacing, de‐escalation, biologics

## Abstract

**Background and Objectives:**

De‐escalation strategies of biologics in psoriasis treatment are widespread in clinical practice. Dose spacing consists of de‐escalating the time range between biological drug injections.

**Patients and Methods:**

Major objectives were: to describe trends in mean PASI, PASI 100, 90, and ≤ 1 from baseline to 12 months after dose spacing, provide drug survival analysis of dose‐spaced regimens, and concurrently describe phenotypic characteristics related to the selection of patient candidates for therapeutic dose spacing. A pre‐post analysis was made between mean PASI at dose spacing and baseline, and after 3, 6, 9, and 12 months following dose spacing.

**Results:**

Of 1,144 patients treated with IL‐23 or IL‐17 inhibitors, 61 patients underwent dose spacing. They presented with lower mean baseline Body Mass Index (BMI) (p = 0.011) and PASI (Psoriasis Area Severity Index) (p = 0.044) and were more frequently bio‐experienced (p = 0.033). 12 months after dose spacing 42.9%, 85.7%, and 92.9% of observed patients achieved PASI 100, 90, and ≤1. There were no significant differences in mean PASI between dose spacing and subsequent time points. The dose spacing survival was 70% at 1 year.

**Conclusions:**

Therapeutic modulation, such as dose spacing, is an effective strategy for most psoriasis patients, resulting in a clear or almost clear skin response that is maintained over time.

## INTRODUCTION

Psoriasis is an inflammatory skin disease characterized by erythematous scaly plaques or patches involving extensor and palmoplantar surfaces of the body, scalp, and nails.^1^ Worldwide, an estimated 125 million people report psoriasis, with a prevalence of 0.5% in Asian countries and up to 8% in some European nations. A mild form of psoriasis can be managed by topical treatments such as topical corticosteroids, vitamin D analogs, steroids plus vitamin D analog combinations, calcineurin inhibitors, and keratolytics.^2^ Phototherapy (narrow‐band UVB or PUVA) can be a suitable option for moderate psoriasis, while traditional systemic treatments such as methotrexate, cyclosporine, and acitretin remain widely used as first‐line therapies for moderate‐to‐severe psoriasis.^2^

Biological drugs have demonstrated high efficacy and safety in the treatment of moderate‐severe psoriasis over the past two decades.^3^, ^4^ Recently, interleukin (IL)‐17 and IL‐23 inhibitors have enabled the achievement of previously inconceivable outcomes such as the *Psoriasis Area Severity Index* (PASI) 90, and 100, and an important impact on quality of life such as the *Dermatology Life Quality Index* (DLQI) 0/1.^3^ In recent years, the possibility of modulating the treatment regimen in terms of dose reduction (de‐escalation) or dose augmentation (escalation) has been of increasing interest.^5^

Possible de‐escalation strategies include reducing the single therapeutic dose, reducing the mg/kg ratio or the number of injections, or dose spacing (D‐S), i.e. extending the interval between administrations.^5^ De‐escalation strategies, although widely used in Europe, are off‐label for the treatment of psoriasis, whereas rheumatology guidelines allow modulation of biological therapy according to clinical response.^5^ Moreover, real‐life experience appears to support de‐escalation for other biological treatment, for instance dupilumab in severe atopic dermatitis.^6^, ^7^

De‐escalation in long‐responder or super‐responder patients would allow an important reduction in healthcare costs, which, although varying from country to country, are a major burden in the healthcare expenditure of countries with universalistic national services.^5^ Furthermore, the reduction of injections over the course of the year could reduce the psychological burden in psoriasis patients, increase compliance, and credibly lead to a reduction in possible adverse events.^5^ Phase II studies have already investigated the efficacy of different dosage regimens of the various biological drugs in a treatment‐naïve psoriatic population.^8^, ^9^, ^10^ Real‐life studies on posological de‐escalation of biologic drugs in psoriatic patients focus particularly on anti‐TNFα therapies, with mixed results.^11^ Data regarding dose de‐escalation, in particular dose spacing, on IL‐23 and IL‐17 inhibitors are lacking to date.^12^, ^13^

With the present study, we attempt to demonstrate the applicability and effectiveness of therapeutic modulation of IL‐17 and 23 inhibitors in psoriatic patients who are long‐responders to these treatments.

## PATIENTS AND METHODS

The present pilot study is a cohort study with retrospective analysis of the general characteristics and efficacy outcomes of psoriatic patients undergoing therapeutic biologic dose spacing, for any reasons, compared to patients undergoing a conventional biologic drug regimen. All patients over 18 years of age undergoing treatment with biologic drugs– IL‐23 inhibitors (guselkumab, risankizumab, tildrakizumab), IL‐17 inhibitors (ixekizumab, secukinumab, brodalumab), and anti‐TNFα therapy (adalimumab) – who were followed at the Dermatology Clinic of the University of Turin from January 2017 to December 2022 were enrolled.

*Primary objectives*:

1. Descriptive analysis in observed cases of trends in mean PASI, PASI 100, PASI 90, and PASI ≤ 1 at baseline (date of biological start) at 16 weeks, at the time of dose spacing, and at 3, 6, 9, and 12 months after dose spacing.

*Secondary objectives*:

1. Drug survival analysis of dose‐spaced regimen.
2. Pre‐post analysis between mean PASI at dose spacing and baseline, and 3, 6, 9, and 12 month time points following dose spacing.

Furthermore, we describe phenotypic characteristics related to the selection of patients who were candidates for therapeutic dose spacing, compared to the non‐regimen modified population. The following were considered: mean age, mean age of onset of psoriasis, mean BMI, gender, involvement of difficult sites (scalp, nails, genitalia, palmoplantar region), joint involvement (PsA), bio‐naive status, mean follow‐up times on treatment with traditional systemic drugs (acitretin, cyclosporine, methotrexate), mean PASI and mean DLQI at the start of biologic treatment.

### Approved drug dose regiment

Traditional, approved regimen for the considered biological agents after initial, specific induction (please refer to specific datasheet):

- Adalimumab 40 mg subcutaneous (SC) injection (inj.) every 2 weeks
- Ixekizumab 80 mg SC inj. every 4 weeks
- Secukinumab 150 mg 2 SC inj. every 4 weeks
- Brodalumab 210 mg SC inj. every 2 weeks
- Guselkumab 100 mg SC inj. every 8 weeks
- Risankizumab 75 mg 2 SC inj. every 12 weeks (150 mg vial. not available at the moment of the study)
- Tildrakizumab 100 mg SC inj. every 12 weeks

### Statistical analysis

Continuous variables were described by mean ± standard deviation (SD) or median and range, based on the distribution of each variable. For categorical variables, absolute and relative frequencies were provided. Percentages were based on the number of non‐missing values. Univariate linear regression (Student's t‐test for continuous variables and chi‐square test for categorical variables), followed by a mixed‐effects logistic regression model (for values with p < 0.2), was employed to investigate potential factors affecting dose‐spacing application. To analyze the patients' dose‐spacing discontinuation of modulated dose regimens, survival analysis techniques were employed. Univariate linear regression (Student's t‐test) was employed for pre‐post analysis. The event was defined as dose‐escalation discontinuation for any reason, while the observation time was calculated from the date of dose spacing to the last follow‐up visit. Statistical analysis was conducted using STATA 15.1 SE (StataCorp., 2017). All tests were two‐sided, and statistical significance was set at α = 0.05.

The present study was approved by our Institutional Review Board under protocol SS‐Dermo‐20.

## RESULTS

A total of 61 out of 1,144 patients (5.3%) experienced therapeutic dose spacing. Compared to the 1,082 patients who did not de‐escalate biologic treatment, the dose‐spaced population at univariate analysis had a lower age (55 vs. 51 years, p = 0.048), a lower mean BMI (24.8 vs. 27.1, p<0.001), a lower mean PASI at the start of biologic therapy (12.2 vs 14.5, p = 0.011) and were more frequently biologic naive (82.5% vs. 64.6%, p = 0.005). Age of onset of psoriasis, mean time on traditional systemic DMARDs (disease‐modifying antirheumatic drugs) treatment, mean DLQI at the start of biologic therapy, being super‐responders, gender, and involvement of difficult sites and joints were not different in the two populations (Table 1). In multivariate analysis, higher BMI (OR 0.9, CI 0.83–0.98, p = 0.011), naive status (OR 0.26 IC 0.1–0.6, p = 0.003), and mean PASI at the start of therapy (OR 0.94, IC 0.88–0.99, p = 0.044) were the only three factors negatively associated with treatment lengthening, partly contradicting the finding in univariate analysis (Table 1).

**TABLE 1:** Univariate analysis of general characteristics of a dose de‐escalated population vs a standard dose population. Multivariate analysis of possible patients’ characteristics associated to dose de‐escalation regimen selection.

	Dose spaced	Standard dose	p value
Total n (%)	61 (5.3%)	1083 (94.7%)	0.602
Mean Age (SD)	51 (16)	55 (15.7)	0.048
Sex (Male) n (%)	38 (62.3%)	710 (65.6%)	0.602
Mean BMI (SD)	24.8 (3.4)	27.1 (5.5)	< 0.001
Mean Age of onset (SD)	36.8 (15.8)	34.9 (15.5)	0.396
Difficult‐to‐treat areas n (%) (1,039 patients)	39 (69.6%)	765 (77.8%)	0.155
PSA n (%) (1,140 patients)	13 (22.8%)	316 (29.2%)	0.301
Bio‐naive n (%) (1137 patients)	47 (82.4%)	695 (64.4%)	0.005
FU under traditional systemic drugs. Mean (SD)	24.1 (21.2)	25.2 (31.5)	0.878
Mean baseline PASI (SD)	12.2 (5.7)	14.5 (6.6)	0.011
Mean baseline DLQI (SD)	20.9 (7.4)	22.5 (7.2)	0.156
Super‐responders n (%)	17 (27.9%)	357 (33%)	0.409
Multivariate analysis			

	*p*	*Odds Ratio*	*95% confidence interval*
Mean Age	0.338	0.99	0.97–1.01
Mean BMI	0.011	0.9	0.83–0.98
Difficult‐to‐treat areas	0.989	0.99	0.44–2.23
Bio‐naive	0.003	0.26	0.1–0.63
Mean baseline PASI	0.044	0.94	0.88 – 0.99
Mean baseline DLQI	0.198	0.97	0.93–1.01

IL‐inhibitors			De‐escalation interruption
IL‐17 inhibitors	41	67.2%	8
IL‐23 inhibitors	20	32.8%	2
Brodalumab	20	32.8%	3
Ixekizumab	11	18%	3
Secukinumab	10	16.4%	2
Risankizumab	9	14.75%	0
Guselkumab	9	14.75%	2
Tildrakizumab	2	3.9%	0

	Baseline	16 weeks	De‐escalation day	3 months after de‐esc	6 months after de‐esc	9 months after de‐esc	12 months after de‐esc	Baseline vs de‐esc	De‐esc vs 3 months after	De‐esc vs 6 months after	De‐esc vs 9 months after	De‐esca vs 12 months after
Mean PASI (SD)	12.2 (5.7)	1.6 (2.1)	0.7 (0.7)	1 (1.5)	1.2 (1.7)	1 (0.5)	0.6 (0.5)	< 0.001	0.209	0.07	0.19	0.899
PASI 100 (%)	0	34.6%	27.9%	31%	33.3%	25.9%	42.9%					
PASI 90 (%)	0	58.2%	73.8%	71.4%	69.4%	66.7%	85.7%					
PASI ≤ 1	0	61.8%	91.8%	83.3%	75%	81.5%	92.6%					

*Abbr*.: SD, standard deviation; BMI, body mass index; PSA, psoriatic arthritis; FU, follow‐up; PASI, Psoriasis Area Severity Index; DLQI, Dermatology Life Quality Index; IL, interleukin; de‐esc, de‐escalation day

Patients’ distribution among class of interleukin inhibitors, and specific treatment. Number and percentage of dose de‐escalation interruption among different treatments.

Mean PASI trend on observed de‐escalated patients at the different time‐points (baseline, 16 weeks, de‐escalation day, 3, 6, 9, and 12 months after de‐escalation). Pre‐post analysis between baseline and de‐escalation day, and between de‐escalation day and following time‐points.

Achievement of PASI100, 90 and ≤ 1 at each time points in observed cases of the same population.

A total of 41 patients dose spaced an IL‐17 inhibitor (67.2%) and 20 an IL‐23 inhibitor (32.8%) (p = 0.036). Of the former, 20 patients (32.8%) spaced brodalumab: 15 patients adjusted to 1 vial SC of 210 mg every 3 weeks, one of whom subsequently switched to 1 vial SC every 4 weeks after 6 months; five patients adjusted to 1 vial SC every 4 weeks, one of whom subsequently switched to 1 vial SC every 8 weeks after 6 months. A total of nine patients (14.8%) spaced guselkumab, all adjusting to 1 vial SC of 100 mg every 12 weeks, with one patient switching to 1 vial SC every 16 weeks after 6 months due to response. Eleven patients (18%) spaced ixekizumab, all of them to 1 vial SC of 80 mg every 6 weeks. Ten patients (16.4%) spaced secukinumab to 2 vial SC of 150 mg every 6 weeks; subsequently, 1 patient further extended the interval to 2 vial SC every 8 weeks. Two patients spaced risankizumab to 75 mg, administered as 2 vial SC every 24 weeks, and 7 patients to 2 vial SC every 16 weeks. Two patients (3.3%) spaced tildrakizumab to 1 vial SC of 100 mg every 16 weeks (Table 1). Dose lengthening occurred on average after 22.6 months (SD 15.2; min. 3–max. 70; median 19.5; Q1–Q3 12–28.3) following the initiation of the spaced biologic drug.

**FIGURE 1:**
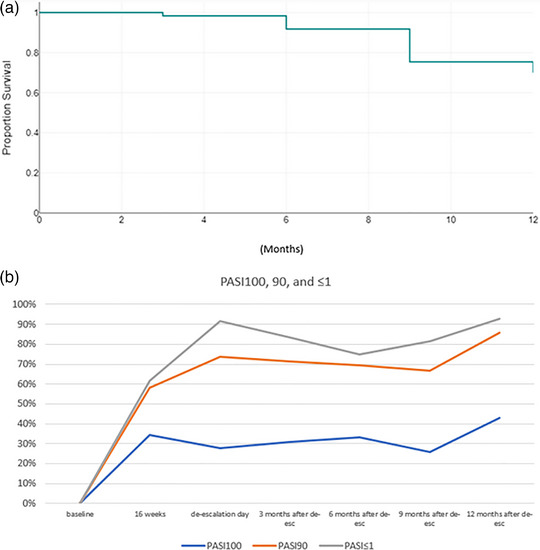
(a) Kaplan‐Mayer curve describing survival at 12 months of dose de‐escalated regimen in the observed population. (b) PASI 100, PASI 90, and PASI ≤ 1 achievement by observed patients at 16 weeks, de‐escalation day, and the following 3, 6, 9 and 12 months.

Overall, in the 61 patients who underwent dose spacing, the initial mean PASI at the start of the full regimen therapy had decreased from 12.2 (SD 5.7) to 1.6 (SD 2.1) at 16 weeks, and at the time of spacing the value was 0.7 (SD 0.7), remaining essentially stable in the observed cases up to 12 months after the change in dosage (0.6, SD 0.8), with a slight worsening at 6 months (1.2, SD 1.7). The PASI 100 at the time of dose spacing was reached by 27.9% with subsequent improvement up to 6 months after dose spacing at 33.3%, up to 42.9% at 12 months in the observed cases. A slight worsening of the outcome occurred at 9 months (25.9%). A similar trend was seen for PASI 90 with 73.8% having reached it at dosing extension, a slight decrease at 9 months (66.7%), up to 85.7% of the observed cases at 12 months. Concerning minimal residual disease PASI ≤ 1, at dose spacing 91.8% of patients had a PASI ≤ 1, with a subsequent worsening of the outcome at 6 months (75%), and an achievement of 92.9% at 12 months. Overall, of the 56 patients who had achieved PASI ≤ 1 at dose spacing, three patients had lost response at 3 months, seven at 6 and 9 months, and eight at 12 months (14.3%) (Table 1, Figure 1a).

Ten patients (16.4%) discontinued dose spacing and returned to conventional dosing, with overall survival of treatment modulation estimated by Kaplan‐Mayer analysis of 70% at 1 year (Table 1, Figure 1b).

In pre‐post analysis, except for the mean PASI at treatment start and dose spacing (12.2 vs. 0.7, p < 0.001), there were no significant differences in mean PASI between dose spacing and subsequent time‐points (3, 6, 9, 12 months) (Table 1).

## DISCUSSION

Only a small proportion of our psoriatic population undergoing treatment with biological drugs underwent therapeutic extension. On the other hand, therapeutic modulation was carried out after almost 2 years of treatment on average, reducing the number of candidate patients among our psoriatic population due to the reduced number of observed cases at extreme time points.

IL‐17 inhibitors were the most dose‐spaced biological drugs, particularly brodalumab. Possible reasons for this could be the large number of patients who have been on these drugs for longer, having been approved earlier for IL‐23, and the high scheduled number of year‐doses, which together with adalimumab is the only drug to have a therapeutic latency of less than 1 month. The high BMI, and mean PASI at the start of treatment have a negative impact on candidacy for treatment extension. The mean PASI at the date of dose spacing was very low, with PASI 90 achieved by more than 70% of patients, though only a small percentage had achieved complete clearance (27.9%). This finding relates to the retrospective nature of the study which included any patient who had spaced the dose, without a defined clinical strategy.

Over the year following prolongation, a minimal overall worsening of outcomes was observed between 6 and 9 months. However, only ten patients returned to standard dosing, with an estimated survival of 70% at 1 year, and no substantial differences were observed between treatments. The achievement of minimal residual disease remained above 90% at all endpoints, although we must report an overall worsening of the picture during dose spacing in at least 14% of patients.

In the literature, most of the real‐life studies focus on the modulation of anti‐TNFα (adalimumab, etanercept, infliximab), data on secukinumab and brodalumab can only be retrieved from phase II/III studies.^11^, ^14^, ^15^ The selection of the psoriatic patient candidate for therapeutic de‐escalation is not homogeneous among the various works, some required the achievement of PASI 100, others required only PASI < 8, and still others like the work of Ataly et al. used PASI < 5 and DLQI < 5.^16^, ^17^, ^18^

Maintenance of these outcomes ranged from 3 months to more than a year before dose spacing was experienced.^11^ Spacing strategies in the literature were variable, adalimumab was administered every 3 to 6 weeks, infliximab every 9 to 11 weeks, ustekinumab up to one injection every 24 weeks, brodalumab from 4 to 6 weeks.^11^ Adalimumab was effective after de‐escalation in 100% of the patients analyzed by Fotiadou et al., but only in 44% of the patients studied by van Bezoojen et al.^17^, ^19^ Similarly, etanercept showed efficacy ranging from 23% to 100%, infliximab from 75% to 100%, ustekinumab from 22% to 85%, secukinumab up to 85.7%, brodalumab maintained a PGA (Physician's Global Assessment) of 0/1 in 12.3% and 5.3% of the patients who had extended to one injection every 4 and 8 weeks, respectively, at week 52.^11^ The assessment of skin worsening also differed in the various papers from loss of PASI < 8 to loss of PASI 100.^17^, ^19^ Michielsen et al. in a review concluded against the therapeutic prolongation of infliximab following an increase in reported adverse events. In the same paper, a non‐substantial increase in anti‐drug antibodies in patients undergoing dose spacing of adalimumab was reported, in partial contrast to what was recently stated by our group.^5^, ^11^ We proposed to select patients eligible for dose spacing among bio‐naive super‐responders (PASI 100 at 6 months), with maintenance of outcomes for at least 6 months, and without features such as joint and special site involvement, high BMI, and metabolic syndrome.^5^

More recently, Gisondi et al. proposed an on‐demand treatment for risankizumab in 64 patients who reported a complete response (PASI 100) after the first three injections (induction phase), the patients resumed the drug only at the onset of a PASI > 1. The mean time between three and 14 injections was 32 weeks, compared to twelve with the conventional regimen, rising to 34 and 39 at the next two injections.^12^

Schots et al., in their real‐life experience with IL‐17 inhibitors, reported a median time of 69.7 weeks before modulation or therapeutic modification, without differentiating between de‐escalation, escalation, switch, or drug interruption. In their study, a total of 18.7% of patients (25) underwent therapeutic modulation: 16 escalated, while nine de‐escalated (6.7%). Of the nine de‐escalated patients two returned to a conventional regimen.^12^ The authors introduced into the work the opportunity to select patients eligible for therapeutic modulation not by clinical or response characteristics, but by therapeutic drug monitoring using blood sample analysis. In the case of a patient with a level of biological drugs in the blood above the range of normality, concomitant with a persistent therapeutic response, de‐escalation could be an appropriate strategy.^13^

Clinical criteria of relapse following dose spacing have been uniquely identified for adalimumab, specifically high BMI, whereas in contrast, male gender and being super‐responders are associated with deferred regimen maintenance.^11^, ^20^, ^21^

The average savings reported in some papers range from 13% to 19% annually.^11^ These results appear low when compared to the savings produced by dose spacing dupilumab in the treatment of patients with severe atopic dermatitis, recently estimated by Spekhorst et al. to be over 30%.^7^

The limitations of our experience, as well as those of the studies presented, include the high heterogeneity of the characteristics and outcomes considered, the low number of patients analyzed, and the inherent constraints of real‐life studies.

Notably, all patients who spaced the dosage were included in the study, which, on the one hand, allows for the identification of baseline patient characteristics but, on the other hand, limits considerations regarding possible clinical strategies for patient selection. From our point of view, even a patient who has not reached full therapeutic response (PASI 100) but has shown some stability of response may be a candidate for dose spacing.

In our work, only two patients prolonged the dosage of tildrakizumab, not allowing us to draw conclusions on the applicability of this strategy on this drug, and in general IL‐23 inhibitors are poorly represented in our work. Although IL‐17 and IL‐23 inhibitors were effective also in rarer variants of psoriasis, dose spacing was conducted only in patients with classic plaque psoriasis.^22^

However, the present study is the first to systematically apply a dose‐spacing strategy to patients treated with an IL‐23 inhibitor and represents one of the largest real‐life experiences involving dose‐spacing de‐escalation of IL‐17 inhibitors. Randomized controlled studies, such as BeNeBio on all IL‐23 and IL‐17 inhibitors and GUIDE on guselkumab, may soon confirm our results.^23^, ^24^

## CONCLUSIONS

Therapeutic modulation, such as dose spacing, is an effective strategy in most psoriasis patients showing a clear or almost clear response of the skin, maintained over time. Future studies are encouraged to evaluate de‐escalation strategies and could help in reducing costs, making the health system more sustainable without substantially impacting the effectiveness of biologic drugs in psoriatic patients.

## FUNDING

The present study was partially funded by UCB pharma.

## CONFLICT OF INTEREST STATEMENT

None.
